# Progesterone from the Cumulus Cells Is the Sperm Chemoattractant Secreted by the Rabbit Oocyte Cumulus Complex

**DOI:** 10.1371/journal.pone.0003040

**Published:** 2008-08-22

**Authors:** Héctor Alejandro Guidobaldi, María Eugenia Teves, Diego Rafael Uñates, Agustín Anastasía, Laura Cecilia Giojalas

**Affiliations:** Centro de Biología Celular y Molecular, Facultad de Ciencias Exactas, Físicas y Naturales, Universidad Nacional de Córdoba, Córdoba, Argentina; Ecole Normale Supérieure de Lyon, France

## Abstract

Sperm chemotaxis in mammals have been identified towards several female sources as follicular fluid (FF), oviduct fluid, and conditioned medium from the cumulus oophorus (CU) and the oocyte (O). Though several substances were confirmed as sperm chemoattractant, Progesterone (P) seems to be the best chemoattractant candidate, because: 1) spermatozoa express a cell surface P receptor, 2) capacitated spermatozoa are chemotactically attracted *in vitro* by gradients of low quantities of P; 3) the CU cells produce and secrete P after ovulation; 4) a gradient of P may be kept stable along the CU; and 5) the most probable site for sperm chemotaxis *in vivo* could be near and/or inside the CU. The aim of this study was to verify whether P is the sperm chemoattractant secreted by the rabbit oocyte-cumulus complex (OCC) in the rabbit, as a mammalian animal model. By means of videomicroscopy and computer image analysis we observed that only the CU are a stable source of sperm attractants. The CU produce and secrete P since the hormone was localized inside these cells by immunocytochemistry and in the conditioned medium by enzyme immunoassay. In addition, rabbit spermatozoa express a cell surface P receptor detected by western blot and localized over the acrosomal region by immunocytochemistry. To confirm that P is the sperm chemoattractant secreted by the CU, the sperm chemotactic response towards the OCC conditioned medium was inhibited by three different approaches: P from the OCC conditioned medium was removed with an anti-P antibody, the attractant gradient of the OCC conditioned medium was disrupted by a P counter gradient, and the sperm P receptor was blocked with a specific antibody. We concluded that only the CU but not the oocyte secretes P, and the latter chemoattract spermatozoa by means of a cell surface receptor. Our findings may be of interest in assisted reproduction procedures in humans, animals of economic importance and endangered species.

## Introduction

Sperm chemotaxis is the oriented movement of the male gamete towards a female attractant source [1]. In mammals, this particular sperm behavior observed in humans, mice, rabbits [1] and recently in bulls [2], is elicited by a small sperm subpopulation (∼10%) corresponding to those cells that completed the capacitation process—a state that enable the spermatozoa to fertilize the egg [1].

For the occurrence of sperm chemotaxis *in vivo*, the egg microenvironment should provide a source of attractants and a stable long lasting attractant gradient [1]. Many sources of chemoattractants were identified in the female reproductive tract such as follicular fluid (FF), oviduct fluid, and conditioned medium from the cumulus oophorus (CU) and the oocyte (O) [1], where several substances were confirmed as sperm chemoattractants [1]. The odorant essences, bourgeonal and lyral chemoattractants, were not found yet in the egg microenvironment. Other chemotactic molecules like atrial natriuretic peptide, small peptides, RANTES chemokine and progesterone (P) are natural components of the FF [1]. However, at the moment of ovulation very tiny amounts of FF are released [3], and a stable long lasting chemoattractant gradient along the oviduct lumen may probably be disrupted by the oviduct contractions.

On the contrary, the viscosity of the cumulus hialuronic matrix provides an environment more resistant to oviduct contractions, where a chemoattractant gradient could be kept stable along the time [4]. In addition, after ovulation the cumulus cells continuously synthesize and secrete the sperm chemoattractant progesterone [5]–[8] and its carrier protein [9] which makes P soluble once out of the cell. Moreover, the CU structure may favor a stable P gradient along the OCC, as we recently suggested [4]. Near the oocyte, the CU secreting P cells are closer each other, hence the quantity of P is probably higher than in the OCC periphery where the CU cells are more isolated. These observations sustain the possibility that the CU and/or its surroundings could be a potential place for P-mediated sperm chemotaxis *in vivo* [4].

Assuming that in mammals: 1) spermatozoa express a cell surface P receptor [10], [11], 2) capacitated spermatozoa are chemotactically attracted *in vitro* by gradients of low quantities of P [12]; 3) the CU cells produce and secrete P after ovulation [5]–[8]; 4) a gradient of P may be kept stable along the CU [4]; and 5) the most probable site for sperm chemotaxis *in vivo* could be along and/or around the CU [1], we postulate that the P secreted by the cumulus cells is the chemoattractant responsible to guide the spermatozoa towards the oocyte.

To test our hypothesis, we carried out experiments in the rabbit, as a mammalian animal model, in order to inhibit the chemotactic response towards the OCC conditioned medium by three different approaches: 1) P from the OCC conditioned medium was removed with an anti-P antibody, 2) the attractant gradient of the OCC conditioned medium was disrupted by a P counter gradient, and 3) the sperm P receptor was blocked with a specific antibody. We concluded that only the CU cells but no the oocyte secret P, which chemoattract spermatozoa by means of a cell surface receptor. Since P is rather conserved along vertebrates, our findings may be of interest in assisted reproduction procedures in humans, animals of economic importance, and endangered species.

## Results

### The cumulus cells are a stable source of sperm chemoattractants

**Figure 1:**
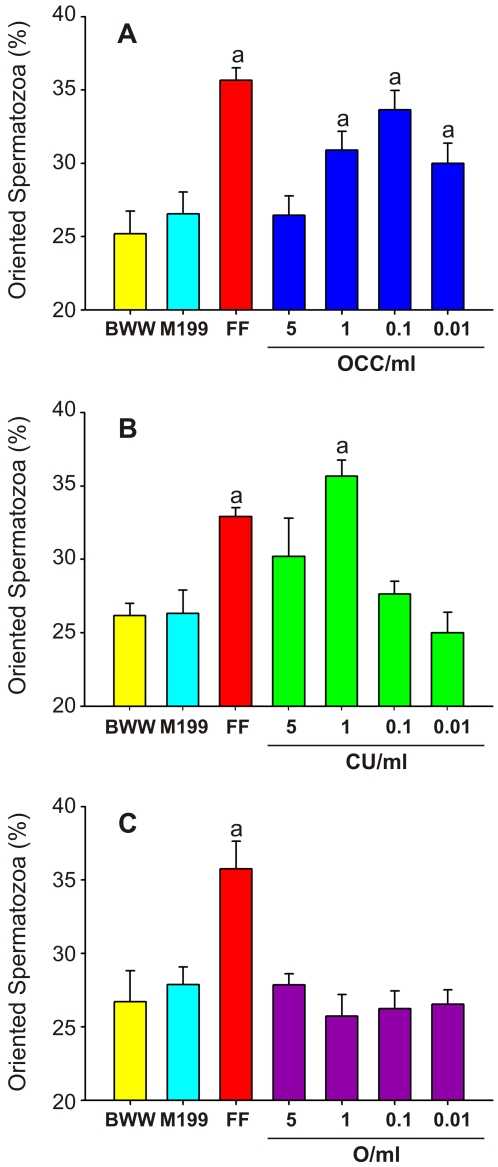
The cumulus cells are the source of sperm chemoattractants. Percentage of oriented spermatozoa towards the OCC (A), CU (B) and O (C) conditioned medium. The sperm capacitation (BWW) and the female cell culture (M199) media were assayed as negative chemotaxis controls, while 1∶10^4^ bovine FF was used as positive chemotaxis control. Four to seven experiments were carried out, giving a total of 600–1,050 analyzed spermatozoa per treatment. Data are expressed as mean±SE. ^a^ Significant differences vs. BWW (*p*<*0.05*).

In humans, a chemotactic activity in the cumulus and the oocyte conditioned media was observed [13]. We first verified whether rabbit spermatozoa were able to chemotactically respond towards conditioned medium from the OCC or its isolated components (the oocyte and the cumulus cells). When spermatozoa were confronted to OCC conditioned medium, a bell-shaped response (typical of any chemotactic cell behavior [14]; Fig. S1) was observed, with a maximum value at 0.1 OCC/ml in comparison to the negative control (Fig. 1A).The chemotactic signal was elicited by a subpopulation of spermatozoa (∼8%, measured as the difference with the BWW negative control), and at almost the same magnitude shown by the FF positive control (Fig. 1A). This responding sperm subpopulation is probably constituted by capacitated spermatozoa, since the level of induced acrosome reacted spermatozoa was similar (8±1%).

We next examined which component of the OCC produces the chemoattractant. A significant chemotactic response towards 1 CU/ml was observed, and at a similar magnitude than the FF positive control (∼10% and ∼8%, respectively; Fig. 1B). As expected, the maximum attractant signal observed in the CU conditioned medium was shifted to a higher concentration, due to the loss of CU cells during OCC manipulation. However, none of the O dilutions tested induced a chemotactic response in rabbit spermatozoa (Fig. 1C).

In addition, there was not a significant chemotactic response towards the M199 medium which was used to culture the female cells, meaning that this medium *per se* did not contain chemoattractants. Besides, no variations in the sperm velocity or in the pattern of movement were observed in any treatment (data not shown). Thus, the proportion of oriented spermatozoa was not influenced by sperm chemokinesis or a hyperactivated behavior. These results suggested that the cumulus cells are a source of at least one chemoattractant.

### The cumulus cells produce and secrete P

**Figure 2:**
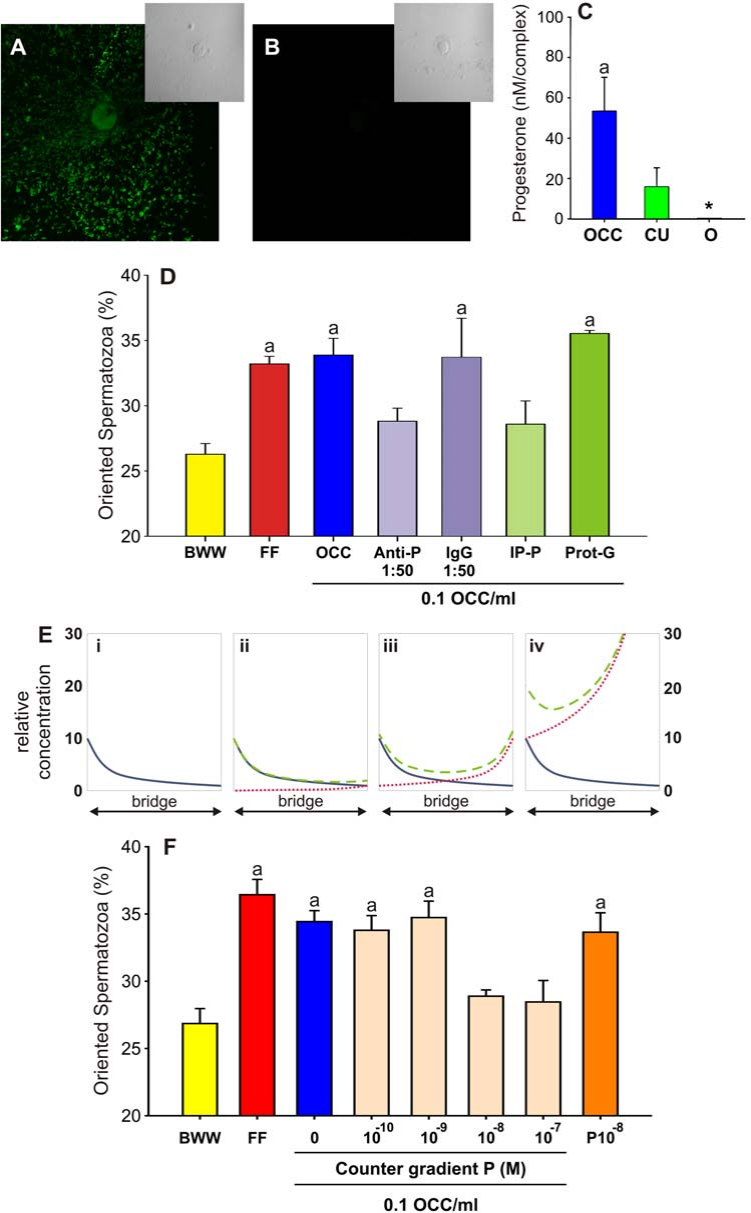
Progesterone is the sperm chemoattractant secreted by the cumulus cells. A and B, Progesterone localization in the OCC by inmunocytochemistry with or without an anti-P antibody, respectively; the corresponding phase contrast images are shown in the insets. Three experiments were carried out. C, Progesterone concentration in the OCC, CU and O conditioned media by enzyme immunoassay. Three experiments were carried out. D, Percentage of oriented spermatozoa towards OCC (0.1 OCC/ml) incubated with an anti-P antibody (Anti-P) or after P immunoprecipitation (IP-P), whereas the unrelated IgG antibody and the protein-G agarose (Prot-G) were assayed as the corresponding negative controls. The sperm capacitation medium (BWW) and 1∶10^4^ bovine FF were assayed as negative and positive chemotaxis controls, respectively. Three to seven experiments were carried out, giving a total of 450–1,050 analyzed spermatozoa per treatment. E, Counter gradient rationale of a theoretical chemotaxis assay, where the relative attractant concentration in the chamber bridge is represented. The chemotactically active dilution of OCC conditioned medium is placed in the well at the left of the chamber bridge, while different dilutions of P are placed at the right side of the bridge. Thus, two opposite one dimension gradients are formed at the same time over the bridge, whereas the OCC attractant diffuses from left to right and the P counter gradient diffuses from right to left. The sperm swimming direction depends on the changes that both attractant gradients may follow after interacting one each other, whereas different possibilities may take place if P is the OCC chemoattractant or it is not. In the case that P is the OCC attractant: i, in the absence of a P counter gradient the spermatozoa swim towards the source of the OCC attractant (solid blue line); ii, the concentration of the P counter gradient is suboptimal (doted red line), then, the spermatozoa swim towards the source of the OCC attractant because the resulting gradient (dashed green line) is similar to the OCC attractant gradient (solid blue line); iii, the concentration of the P counter gradient is equivalent to that of the OCC, therefore the resulting gradient (dashed green line) is symmetric and the spermatozoa swim at random; iv, the concentration of the P counter gradient (dotted red line) is higher than that of the OCC attractant, the resulting gradient (dashed green line) is asymmetric, but the sperm P receptors are saturated and the cell swim at random. In the case the OCC attractant is not P, the sperm swimming direction varies according to the concentration of the P counter gradient (dotted red line): at suboptimal P concentration the spermatozoa swim towards the OCC source of attractant (ii), at optimum concentration the spermatozoa swim at random due to an attractant choice conflict (iii), and at saturated concentration the spermatozoa swim towards the OCC source (iv). In summary, to confirm that P is the OCC chemoattractant, an inhibition of sperm chemotaxis should be observed under two different conditions: when the P concentration is similar in both opposite gradients (iii), and when P is at a higher concentration in the counter gradient (iv); since, only the first condition (iii) does not allow to clarify the identity of the OCC attractant. F, Sperm chemotaxis towards the OCC conditioned medium (0.1 OCC/ml) containing ∼0.5×10^−8^ M P, was inhibited by a counter gradient of P with a similar (10^−8^ M) or higher (10^−7^ M) steroid concentration. The sperm capacitation medium (BWW), 1∶10^4^ bovine FF and 10^−8^ M P were assayed as negative (the first one) and positive (the latter two) chemotaxis controls. Five to eight experiments were carried out, giving a total of 750–1,200 analyzed spermatozoa per treatment. Data are expressed as mean±SE. ^a^ Significant differences vs. BWW (*p*<*0.05*). * Not detected.

We previously reported that the distribution of the CU cells secreting P favor a stable P gradient in human OCC [4], then we next verified whether rabbit OCC was also able to produce and secrete P to the medium. Progesterone was observed inside the cumulus cells with an intense green label whereas the oocyte showed less fluorescence (Fig. 2A). In the absence of the primary antibody no cell labeling was observed (Fig. 2B). In order to know whether P was actively secreted by the OCC, we measured the P concentration in the OCC, CU and O conditioned media. In the OCC and CU conditioned media the P concentration was 53±17 nM and 16±12 nM, respectively; but in the O conditioned medium no P was detected (Fig. 2C). These results showed that rabbit CU cells synthesize and secrete P to the medium.

### Inhibition of the sperm chemotactic response towards the OCC conditioned medium

Considering that rabbit CU but not the oocyte secretes chemoattractants, we performed the chemotaxis assays with the OCC conditioned medium in order to preserve its physiological structure. In all the experiments, the dilution which induced the highest chemotactic activity (0,1 OCC/ml) was used. To test whether P was the sperm chemoattractant of the OCC conditioned medium, we carried out three different approaches described below.

#### Removal of P from the OCC conditioned medium

The chemotaxis assay was performed with OCC conditioned medium where P was either previously removed by immunoprecipitation or blocked by an anti-P antibody. The OCC conditioned medium chemotactic activity was significantly decreased after immunoprecipitating P (Fig. 2D), whereas no chemotaxis inhibition was observed in the presence of the precipitating agent (Protein G-agarose complex; Fig. 2D). The OCC chemotactic activity was also significantly inhibited by a 1∶50 dilution of anti-P antibody, whereas 1∶500 and 1∶5000 antibody dilution (data not shown) and an unrelated IgG did not affect sperm chemotaxis (Fig. 2D). Thus, the removal of P from the OCC conditioned medium by specific antibodies abolishes the sperm chemotactic response.

#### A progesterone counter gradient formed in opposition to the OCC attractant gradient

A stable attractant gradient is essential for the occurrence of chemotaxis, which may be disrupted by a simultaneous counter gradient of the same attractant at a similar concentration. The rationale of these experiments is shown in figure 2E. In order to confirm that P is the OCC chemoattractant, an inhibition of sperm chemotaxis must be observed under two different conditions: when the P concentration is similar in both opposite gradients (Fig. 2E, iii); and when P is at a higher concentration in the counter gradient (Fig. 2E, iv); only the first situation (Fig. 2E, iii) does not allow to clarified whether the OCC chemoattractant is P or a different molecule.

When different concentrations of P were loaded in the opposite well to the OCC, the sperm chemotactic response was inhibited under a counter gradient of 10^−8^ M and 10^−7^ M P (Fig. 2F). In the absence of P counter gradient, 10^−8^ M P induced sperm chemotaxis at the same magnitude than the other positive controls (FF and OCC). The P concentration in the OCC chemotactic dilution was ∼0,5×10^−8^ M, which was similar to the 1×10^−8^ M P counter gradient that disrupted the OCC attractant gradient. These results showed that a counter gradient of P inhibits the sperm chemotactic response towards the OCC conditioned medium.

#### Progesterone sperm surface receptor blockage

**Figure 3:**
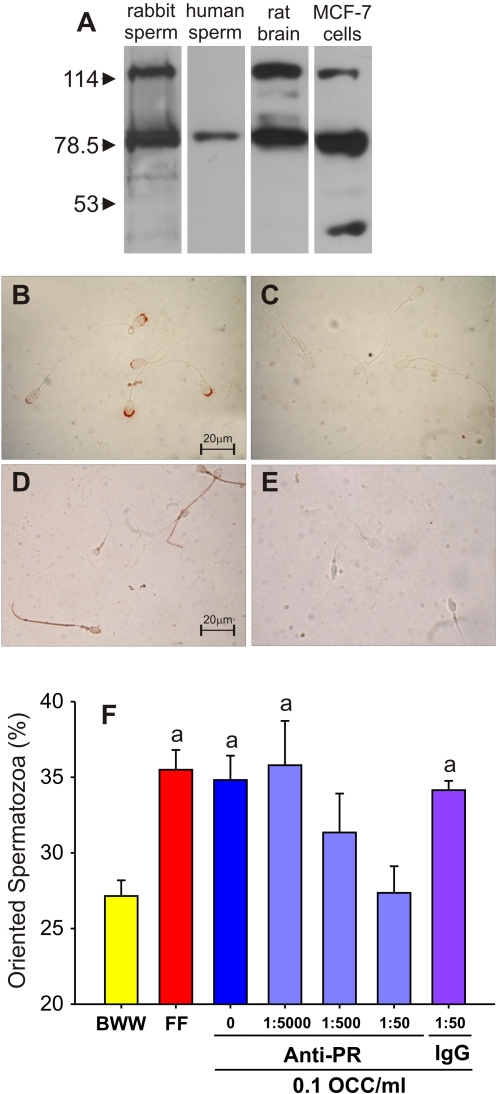
A cell surface P receptor is involved in the sperm chemotactic response. A, western blot with an anti-P receptor antibody in rabbit and human spermatozoa; rat brain and MCF-7 cell line lysates were carried out as positive controls. Three experiments were carried out. B–E, cell surface P receptor localization in non permeabilized spermatozoa by immunocytochemistry with an anti-P receptor antibody. The P receptor is localized over the acrosomal region in rabbit spermatozoa (B), and also in the tail of human spermatozoa (D); C and E are the respective negative controls in the absence of anti-P receptor antibody. F, Percentage of oriented spermatozoa towards the OCC conditioned medium (0.1 OCC/ml) previously incubated with anti-P receptor antibody (Anti-PR), whereas the incubation with an unrelated antibody (IgG) was assayed as negative control. The sperm capacitation medium (BWW) and 1∶10^4^ bovine FF were assayed as negative and positive chemotaxis controls, respectively. Four to five experiments were carried out, giving a total of 600–750 analyzed spermatozoa per treatment. Data are expressed as mean±SE. ^a^ Significant differences vs. BWW (*p*<*0.05*).

In several mammalian species, a P receptor was observed in the sperm cell surface [10], [11], however, no report was found in the rabbit. We first verified the presence of a P receptor in rabbit spermatozoa by means of western blot and immunocytochemistry. Two proteins of ∼120 kDa and ∼80 kDa MW were detected in rabbit spermatozoa (Fig. 3A). Since the antibody was developed for human breast cancer cells, and it was described that human spermatozoa express a P receptor [10], we also searched for the presence of a P receptor in human sperm lysate, where only a protein of ∼80 kDa was observed (Fig. 3A). Cell lysates from rat brain and the MCF-7 cell line were assayed as positive P-receptor controls [10], [15]. In both cases, the antibody recognized the same MW proteins observed in rabbit spermatozoa, but an additional protein of ∼45 kDa was observed in the MCF-7 cell line. These results suggested the presence of two P-receptors in rabbit spermatozoa. In order to know whether these receptors were located in the cell surface, live capacitated rabbit spermatozoa were incubated with the antibody used for western blot, without cell permeabilization. Rabbit spermatozoa showed an intense label over the acrosomal region (Fig. 3B), whereas in human spermatozoa it was also observed in the tail (Fig. 3D). No labeling was observed when the primary antibody was omitted as a negative control (Fig. 3C and 3E). The results suggested the presence of at least one P-receptor located in the surface of rabbit spermatozoa.

Next, we incubated live capacitated spermatozoa with the antibody against P-receptor, and then carried out the chemotaxis assay against the OCC conditioned medium. Sperm chemotaxis was inhibited in a dose-dependent manner when the cells were previously incubated with increasing concentrations of the anti-P receptor antibody. The incubation of the spermatozoa with a non-related IgG (at the same concentration that the anti-P receptor antibody inhibited sperm chemotaxis) did not abolish the chemotactic response (Fig. 3F). The positive controls (FF and OCC in the absence of the antibody) were significantly higher than the negative control (Fig. 3F). Therefore, when the P receptor is blocked, the sperm chemotaxis towards the OCC conditioned medium does not take place.

## Discussion

In the rabbit, we observed that only the cumulus cells but not the oocyte secretes P, which chemoattracts spermatozoa by means of a cell surface receptor. Since the presence of this steroid in the fertilization site is widely distributed among vertebrates, our findings may be of interest in assisted reproduction procedures for improving fertility in humans, animals of economic importance and endangered species.

### The cells from the cumulus oophorus but not the oocyte are a stable source of sperm chemoattractants

It is well known that FF contains attractants that chemotactically orient the spermatozoa of several mammalian species such as human, mouse, rabbit and bull [1], [2]. Although the quantities of FF that may enter the oviduct lumen during ovulation are negligible [3], some of this viscose and sticky fluid could remain inside the cumulus oophorus. Therefore, our finding of a sperm chemotactic response towards the OCC conditioned medium could be due to the remaining FF. In order to rule out this possibility, we incubated the OCC for 24 h when the cumulus was completely expanded. Thus, any trace of FF chemoattractants was allowed to diffuse to the culture medium which was replaced with fresh medium at the end of that period. The cells were next cultured for 24 h when the conditioned medium was collected for the chemotaxis assays. In the latter OCC conditioned medium we found sperm chemotactic activity which means that the cells continued secreting chemoattractants for at least 48 h. In the rabbit, where ovulation is induced by mating, the fertilization window lasts around 20 h [16], [17]. Therefore, the cells from the OCC are able to ensure a stable source of attractants for a much longer period of time than the required under *in vivo* conditions.

In many marine invertebrates the sperm chemoattractant is secreted by the oocyte (or released from its surrounding jelly), but in some groups the attractant is secreted by the accessory cells that accompany the oocyte [18]. In humans, it was suggested that both the cumulus cells and the oocyte are sources of attractants [13], but in the rabbit we found that the cumulus cells, but not the oocyte, secrete chemoattractants. Under *in vivo* conditions, by the time of ovulation, only those oocytes surrounded by the cumulus cells are fertilized by rabbit spermatozoa [19]. After fertilization, the cumulus is rapidly disorganized [20], thus, the chemoattractant gradient is disrupted and polyspermy could be prevented. These observations support the idea that only the cumulus cells secret the attractant that guides rabbit spermatozoa towards the oocyte.

### Progesterone is the sperm chemoattractant secreted by the cumulus cells

Many substances have been shown to chemotactically guide mammalian spermatozoa, some of them such as bourgeonal and lyral were not yet identified in the female reproductive tract. Instead, the sperm chemoattractants atrial natriuretic peptide, small peptides, RANTES chemokine and P are natural components of FF [1]. From these molecules, P seems to be the best candidate to chemoattract mammalian spermatozoa *in vivo* due to several reasons: 1) spermatozoa express a cell surface P receptor [10], [11], 2) capacitated spermatozoa are chemotactically attracted *in vitro* by gradients of low hormone quantities [4], 3) the cumulus cells continuously produce and secrete P after ovulation [5]–[8], 4) a gradient of P may be kept stable along the CU [4], and 5) the most probable site for sperm chemotaxis *in vivo* could be along and/or around the CU [1]. To test the hypothesis that P is the chemoattractant secreted by the cumulus cells that guides the spermatozoa towards the oocyte, we verified in the rabbit that: 1) the cumulus cells secrete the hormone, 2) the spermatozoa expressed a cell surface P receptor, and 3) the sperm chemotactic response towards the OCC conditioned medium is inhibited by depleting P from the conditioned medium, disrupting the P gradient from the OCC conditioned medium and blocking the sperm P receptor.

#### The cumulus cells supply a long lasting availability of P

Progesterone is synthesized [5] and secreted by the cumulus cells in humans [7], mice [6] and pigs [5]. Though P participates in the oocyte maturation, it is not synthesized by the oocyte itself but rather it is up taken from its surroundings [5]. By means of immunocytochemistry with an anti-P antibody the hormone was localized inside the cumulus cells and the oocyte, the latter showing a lower fluorescent intensity. Moreover, P was found in the OCC and CU but not in the O conditioned medium, indicating that only the cumulus cells secrete P to the medium, in agreement with the observations in other species [5]–[8]. After 48 h of culturing, the P concentration in the OCC conditioned medium was ∼53 nM per complex, suggesting that the cumulus cells are able to supply a long lasting availabity of the hormone. However, the P concentration in the OCC conditioned medium chemotactic dilution was ∼5 nM, which is an order of magnitude higher than previously reported in the rabbit [12]. Since the cumulus cells also secret a corticosteroid binding globulin which binds P with a higher affinity than the albumin [21], the chemotactic P concentration in the OCC conditioned medium could be even lower.

#### Rabbit spermatozoa express a cell surface P receptor

In mammalian spermatozoa, a cell surface P receptor not completely characterized yet was observed, in association to sperm processes that are promptly triggered by the hormone [22]. Sperm chemotaxis also requires a ligand cell surface receptor because the cell must be ready to change its swimming direction when an attractant concentration gradient is detected [1]. Then, we verified whether rabbit spermatozoa also express a P receptor by means of western blot and immunocytochemistry with a monoclonal anti-P receptor antibody, which was developed against the nuclear P receptor of human breast cancer cells. Two proteins of ∼80 kDa and ∼120 kDa were identified with this antibody in the rabbit sperm lysate; the first one was the only protein observed in human spermatozoa. In non permeabilized live rabbit spermatozoa the P receptor was mainly localized in the acrosomal region, suggesting that at least one of those receptors is localized at the cell surface. Other laboratories reported the presence of cell surface P receptor in human, dog, pig and mouse spermatozoa [10], [23]–[29] detected with different antibodies against the genomic P receptor. However, there is variation in the number of proteins identified (one or two), their molecular weights (from ∼44 kDa to ∼85 kDa), the receptor distribution (acrosomal region, equatorial zone, tail) and the protocols used [10], [23]–[29]. Moreover, a new family of membrane progestin receptor (mPR) of ∼40 kDa was recently described in fish and mammalian spermatozoa [11], enlarging the variety of P receptors. Although there is no doubt that mammalian spermatozoa express a cell surface receptor, its identity is far to be solved. This is mainly due to the fact that the spermatozoon is a haploid highly differentiated cell, transcriptional inactive, with almost no cytoplasm, where some of the main molecular approaches can not be applied. Our results suggested that rabbit spermatozoa express at least one cell surface P receptor, additional studies to further characterize such receptor are needed.

#### Inhibition of the OCC conditioned medium chemotactic activity

When P was either removed by immunoprecipitation or blocked by an anti-P antibody, the sperm chemotaxis towards the OCC conditioned medium was inhibited. In addition, in the presence of a P counter gradient at a concentration similar or even higher than that found in the chemotactic OCC conditioned medium, the sperm chemotactic response was completely inhibited. When rabbit spermatozoa were incubated with the anti-P receptor antibody, the sperm chemotaxis towards the OCC conditioned medium was inhibited in a dose dependent manner. In any event, if the OCC chemoattractant were different from P, sperm chemotaxis should not be suppressed.

Thus, the chemotactic activity of the OCC conditioned medium was inhibited by several different approaches, suggesting that P is the sperm chemoattractant secreted by the CU cells, and that sperm chemotaxis requires a prompt cell response mediated by a cell surface P receptor.

Although this study is mainly concerned with P mediating sperm chemotaxis, the action of this hormone is much more versatile, enhancing a wider number of physiological events in mammalian spermatozoa [30], [31]. Results from this work and previous studies provide evidence to support a model for the interaction of P and spermatozoa under *in vivo* conditions. In the periphery of the OCC the spermatozoa detect a P concentration gradient generated by the cumulus cells, where at low concentrations the hormone would chemotactically guide spermatozoa [12] towards the oocyte, in parallel to priming them for the acrosome reaction (unpublished data); then, while approaching to the oocyte, higher P concentration gradients would induce a hyperactivated movement [12], which in turn, would help the sperm cell to cross the last vestments of the oocyte to finally fertilize it.

## Materials and Methods

All chemicals were from Sigma-Aldrich (St. Lois, MO, USA) unless otherwise indicated.

### Sperm preparation

Rabbit spermatozoa were obtained with an artificial vagina [32] from fertile New Zealand bucks (6–12 month old), in accordance with the Guide for Care and Use of Laboratory Animals [33]. Spermatozoa were separated from the seminal plasma by migration-sedimentation technique [34], in BWW medium [35] supplemented with 40 mg/ml of BSA fraction V and 50 mM Hepes, for 15–20 min at 37°C, under an atmosphere of 5% CO_2_ in air. Then, the sperm suspension was adjusted to 4×10^6^ cells/ml and incubated under the above conditions for 16 h, when the proportion of capacitated spermatozoa *in vitro* [36] and *in vivo* [37] is maximum. Sperm capacitation was indirectly determined as the percentage of induced acrosome reaction by means of A23187 calcium ionophore and the acrosome marker PSA-FITC, as previously described [38].

Human spermatozoa were used as an additional control for P receptor detection. Normal sperm samples (according to the WHO criteria) were separated from the seminal plasma in a discontinuous Percoll gradient as described [39]. The highly motile sperm fraction was incubated in HAM-F10 with 25 mM Hepes and L-Glutamine (Invitrogen, California, USA) supplemented with 1% of HSA (UNC, Argentina) for 4 h at 37°C, under an atmosphere of 5% CO_2_ in air.

### Conditioned medium preparation

Fertile female New Zealand rabbits were induced to ovulate by mating. Before ovulation (8–9 h post-coitus) the ovaries were surgically removed and the OCC were obtained by puncturing mature ovarian follicles (>3 mm). Each OCC was washed 3 times with M199 medium, and then two OCC per well were incubated with 250 µl of M199 in a multiwell plate at 37°C under an atmosphere of 5% CO_2_ in air. After 24 h, an aliquot of 200 µl of the supernatant were replaced with fresh medium, continuing the cell culture for additional 24 h. At the end of the incubation period, the conditioned medium was centrifuged for 10 min at 2,000× g, and the supernatant was kept at −20°C until the day of the experiment. Only the conditioned medium from completed expanded OCC, with a viable oocyte and more than 70% of live cumulus cells (checked with 0,1 µg/ml Hoeschst 33258 [40]) were used in the experiments. In order to obtain the CU and O conditioned media, the isolated OCC were passed several times through a fine-bore glass pipette with 0,1 mg/ml of hyaluronidase. The denuded oocytes were washed 3 times with M199 medium and then placed in a multiwell plate at a rate of two oocytes in 250 µl of M199 medium per well. The cumulus cells were washed two times by centrifugation (150× g for 5 min), and then the cells from two OCC were incubated with 250 µl of M199 medium per well in a multiwell plate. The O and CU conditioned media were processed in the same way than the OCC conditioned medium described above.

### Sperm chemotaxis determination

Chemotaxis assays were performed in a chemotaxis chamber by videomicroscopy and computer image analysis as previously described [38]. A brief description of the rationale of chemotaxis determination is shown in figure S1A. Spermatozoa were recorded at 6 Hz with the Virtualdub software (ver. 1.6.16, Avery Lee; http://www.virtualdub.org/). The sperm tracks were analyzed with the ImageJ software (ver. 1.38, NIH, USA) and the MtrackJ plugin (ver. 1.1.0, Eric Meijering; http://www.imagescience.org/meijering/software/mtrackj/). The percentage of “oriented spermatozoa” was calculated in 150 spermatozoa per treatment with the SpermTrack software (ver. 4.0, UNC, Argentina). A dose response chemotaxis assay with several dilutions of the conditioned media was performed. In parallel, the BWW sperm capacitating medium and the M199 female cell culturing medium were carried out as negative controls. Bovine FF (1∶10^4^) was assayed as positive control since it is able to chemoattract rabbit spermatozoa [41]. The curvilinear velocity and the pattern of movement (linear, transitional or hyperactivated) were determined in 50 spermatozoa per treatment, as previously described [38].

### Progesterone detection in the OCC

After collecting the conditioned medium, some OCC were fixed with 4% paraformaldehyde, and then the cells were permeabilized with 0.2%Triton X-100 and blocked with 1% BSA in PBS. The OCC were incubated with rat anti-P primary antibody (1∶50) overnight at room temperature. After washing, the samples were incubated with an anti-rat FITC-conjugated secondary antibody (1∶50). The preparations were fixed with 0.4% paraformaldehyde and mounted in a coverslip with an anti-bleaching solution (Dako, Denmark). The same procedure was followed for the negative control but in the absence of the primary antibody. The preparations were observed under a confocal microscope (LSM 5 Pascal, Zeiss, Germany).

### Progesterone determination in the conditioned media

Conditioned media were diluted 1∶10 in human male serum and the P concentration was measured by means of a solid- phase competitive chemiluminescent enzyme immunoassay, in an IMMULITE 2000 autoanalyzer (Siemens, Germany), with a sensitivity of 0,1 ng/ml. The P concentration was calculated by discounting the blank sample (M199 medium), previously diluted (1∶10) in human male serum.

### Progesterone receptor detection in spermatozoa

#### Western Blot

An aliquot of capacitated spermatozoa was centrifuged at 1,500× g for 5 min and the pellet was resuspended at 6×10^6^ cells per 10 µl of radio immunoprecipitation assay (RIPA) buffer with a protease inhibitors cocktail. The samples were mixed with a 1∶1 solution of Laemmli buffer with 2.5% beta-mercaptoethanol, and boiled for 6 min at 95°C. The proteins were separated by 10% SDS-poliarcylamide gel electrophoresis and then transferred to 0.2 µm nitrocellulose membrane (BioRad, USA). The samples were blocked with 5% dried milk in Tris-buffered saline with 0.1% Tween 20, and incubated with a mouse monoclonal anti-P receptor primary antibody (1∶1,000; Clone HPRA2+Clone HPRA3) for 24 h, followed by the anti-mouse IgG biotin-conjugated secondary antibody (1∶500) and streptavidin-peroxidase (1∶500). Proteins were visualized by enhanced chemiluminescence. Human breast cancer cell line (MCF-7; American Type Culture Collection, USA) and rat brain lysates were used as positive controls for P receptor [10], [15].

#### Immunocytochemistry

Capacitated live spermatozoa were incubated with the mouse monoclonal anti-P receptor primary antibody (1∶25) used for the western blot, for 30 min at 37°C, and then washed with PBS. The samples were fixed with 1% formaldehyde and centrifuged twice at 800× g for 7 min. The resuspended pellet was smeared in a slide and let dry on air at room temperature. After washing the samples with PBS, the endogenous peroxidase activity was blocked with 3% H_2_O_2_, and the cells were incubated with the anti-mouse IgG biotin conjugated secondary antibody (1∶100) for 2 h, followed by streptavidin-peroxidase treatment (Lab Vision, UK). The spermatozoa were observed under a light microscopy by means of the AEC staining kit.

### Progesterone removal from the OCC conditioned medium

Aliquots of the chemotactic OCC conditioned medium (0.1 OCC/ml) were incubated with rat anti-P antibody (1∶50; 1∶500 and 1∶5,000) for 30 min, at room temperature before the sperm chemotaxis assay, where the conditioned medium with a mouse anti-IgG (1∶50) was carried out as negative control. For immunoprecipitation, 100 µl of the OCC conditioned medium (8 OCC/ml) were incubated overnight at 4°C with 10 µl of rat anti-P antibody, and then 10 µl of Protein-G PLUS agarose complex (Santa Cruz Biotechnology, Inc., USA) were added for 5 h. The samples were centrifuged at 5,000× g for 1 h and the supernatant was stored at −20°C until the day of the experiment. The negative control was performed incubating the OCC conditioned medium only with the protein G-agarose complex.

### Statistical analysis

At least three experiments were performed per treatment. Statistical differences between treatments were determined with the ANOVA and the Holm-Sidak tests by means of the SigmaStat software (SPSS, Inc, USA).

## Supporting Information

Figure S1 Chemotaxis assay. A, The chemotaxis chamber consists in two wells separated by 1 mm wall, one filled with medium with or without attractants and the other one with capacitated spermatozoa. The chamber is sealed with a coverslip, thus, a capillary space (called bridge) is formed between both wells and over the separating wall. Across the bridge, a one dimension attractant concentration gradient is formed in the direction of the well containing the spermatozoa, which in turn, swim up over the bridge. Ten minutes after sealing the chamber (time necessary to stabilize the sperm distribution and the attractant gradient), the sperm movement is recorded along the fields in the middle of the bridge. Then, the sperm tracks are analyzed by computer imaging to calculate chemotaxis and other sperm dynamic parameters (e.g., sperm velocity and pattern of movement). B, For each sperm track, the distance traveled along the X axes, (representing the attractant gradient; ΔX) and the Y axes (representing the absence of attractant gradient; ΔY) are calculated. Assuming that a chemotactic spermatozoon travel a longer distance along the X axes than in the Y axes, sperm directionality is calculated by the quotient ΔX/|ΔY|. When the value is >1, the spermatozoon is considered oriented towards the attractant well. As negative control, a buffer without attractants is loaded instead the attractant containing solution. In this case, the spermatozoa swim at random in the bridge; therefore, ∼25% of the cells are expected to be oriented towards the well without attractants. The chemotactic responding subpopulation is considered as the difference in the percentage of “oriented spermatozoa” between the attractant solution and the negative control. C, The chemotactic response is strongly dependent on the attractant concentration; therefore, several doses of the attractant solution should be assayed. The expected chemotaxis result is a bell-shaped curve, typical of any chemotactic cell, where at low concentration there is not enough attractant receptors stimulated, while they are saturated at high attractant concentration. As a consequence, in both extreme cases the chemotaxis response is abolished and the level of “oriented spermatozoa” is similar to the basal negative control (∼25%). At optimum attractant concentration the cells are able to sense the attractant gradient and respond with a chemotactic movement orientation; therefore, the level of “oriented spermatozoa” is expected to be statistically higher than the basal negative control. As in mammals such a difference is ∼10%, a high number of spermatozoa (150) per treatment must be analyzed, in at least three experiments.
(1.07 MB TIF)
